# Estrogen Receptor Beta Influences the Inflammatory p65 Cistrome in Colon Cancer Cells

**DOI:** 10.3389/fendo.2021.650625

**Published:** 2021-03-30

**Authors:** Rajitha Indukuri, Linnea Hases, Amena Archer, Cecilia Williams

**Affiliations:** ^1^ Science for Life Laboratory, Department of Protein Science, KTH Royal Institute of Technology, Solna, Sweden; ^2^ Department of Biosciences and Nutrition, Karolinska Institutet, Huddinge, Sweden

**Keywords:** p65, ERβ, ChIP, colon cancer, colorectal cancer (CRC), estrogen receptor

## Abstract

Inflammation is a primary component of both initiation and promotion of colorectal cancer (CRC). Cytokines secreted by macrophages, including tumor necrosis factor alpha (TNFα), activates the pro-survival transcription factor complex NFκB. The precise mechanism of NFκB in CRC is not well studied, but we recently reported the genome-wide transcriptional impact of TNFα in two CRC cell lines. Further, estrogen signaling influences inflammation in a complex manner and suppresses CRC development. CRC protective effects of estrogen have been shown to be mediated by estrogen receptor beta (ERβ, *ESR2*), which also impacts inflammatory signaling of the colon. However, whether ERβ impacts the chromatin interaction (cistrome) of the main NFκB subunit p65 (RELA) is not known. We used p65 chromatin immunoprecipitation followed by sequencing (ChIP-Seq) in two different CRC cell lines, HT29 and SW480, with and without expression of ERβ. We here present the p65 colon cistrome of these two CRC cell lines. We identify that RELA and AP1 motifs are predominant in both cell lines, and additionally describe both common and cell line-specific p65 binding sites and correlate these to transcriptional changes related to inflammation, migration, apoptosis and circadian rhythm. Further, we determine that ERβ opposes a major fraction of p65 chromatin binding in HT29 cells, but enhances p65 binding in SW480 cells, thereby impacting the p65 cistrome differently in the two cell lines. However, the biological functions of the regulated genes appear to have similar roles in both cell lines. To our knowledge, this is the first time the p65 CRC cistrome is compared between different cell lines and the first time an influence by ERβ on the p65 cistrome is investigated. Our work provides a mechanistic foundation for a better understanding of how estrogen influences inflammatory signaling through NFκB in CRC cells.

## Introduction

Colorectal cancer (CRC) accounts for the third highest number of cancer deaths among both women and men in the Western world (1). Benign adenomatous polyps evolve into carcinomas over 10-15 years, and screening is performed in many countries. While COX-2 inhibitors (e.g. celecoxib) or aspirin reduce inflammation and effectively prevent adenomatous polyp formation and CRC, adverse effects exclude their general usage (2–4). Better preventive treatments are required, and a deeper understanding of exactly how inflammation impacts CRC is needed.

During inflammation, cytokines such as tumor necrosis factor alpha (TNFα) are released by macrophages. TNFα activates the nuclear factor kappa-light-chain-enhancer of activated B cells (NFκB) transcription factor complex, which in turn induces several oncogenes and signaling pathways involved in tumor initiation and progression (5–7). Constitutive activation of NFκB has been observed in nearly 70% of CRC cell lines and 40% of clinical CRC specimens (8–11). NFκB is a homo- or heterodimer comprised of two to five of subunits p65 (RelA/RELA), RelB (RELB), cRel (RELC), p105/p50 (NFκB1), or p100/p52 (NFκB2), that appears in multiple forms (12). The N-terminal Rel domain is present in all subunits and binds to a specific DNA sequence known as the κB site. Together with the recruitment of cofactors, this induces or suppresses expression of target genes. The various NFκB dimers differ in binding affinity and activation. p65 and cRel are the most potent transcriptional activators (13), and p65 together with p50 constitutes the most common NFκB heterodimer (14). While inflammation is critical in development of CRC, the function of NFκB complex in CRC is, however, poorly studied. Only one study describes the genome-wide chromatin binding of p65 in colon cancer, in cell line SW480 in the context of its interaction with p53 (TP53) mutants (15).

The hormone estrogen has been shown to reduce CRC incidence (16–20). Estrogen mainly acts through three receptors, of which estrogen receptor beta (ERβ, *ESR2*) is present in epithelial colon and rectal cells (21, 22). We have recently shown that intestinal epithelial ERβ *in vivo* protects from the epithelial damage caused by TNFα and prevents tumor formation (22). Also, when reintroduced into CRC cell lines, ERβ has antiproliferative and tumor-suppressive activity (22, 23). ERβ is a ligand-activated nuclear receptor which binds to genomic ERE (estrogen response elements). Its homologue, ERα (*ESR1*) is upregulated in breast cancer, where it promotes cell proliferation and interacts with p65 (24, 25). ERβ expression, in contrast, decreases during CRC development, and ERα is not expressed in the colon epithelial cells, nor tumors (26). Our hypothesis is that ERβ in the normal colon opposes NFκB-mediated inflammatory signaling and that this is an essential part of its tumor protective mechanism.

A crosstalk between the related ERα and NFκB has been extensively studied in breast cancer, albeit with some contradicting findings. A few studies report that ERα represses NFκB activity (27, 28), whereas other reports that ERα, in the same cell lines (MCF7, T47D, ZR-75), enhances NFκB activity (24, 25). Specifically, TNFα in ERα-positive MCF-7 breast cancer cells was shown to profoundly modify the ERα enhancer-binding landscape in an NFκB-dependent manner (29). Based on the homology between the two ERs DNA-binding domains, along with previous findings that ERβ regulates NFκB key targets and reduces inflammatory signaling in colon, we speculated that ERβ may impact the colon p65 cistrome. In the present study, we used p65 chromatin immunoprecipitation (ChIP) followed by sequencing (ChIP-Seq) to test this hypothesis, and to detail the p65 landscape in CRC cell lines.

## Materials and Methods

### Cell Culture

SW480 (Research Resource Identifier RRID: CVCL_0546) and HT29 (RRID: CVCL_0320), previously generated to express ERβ and corresponding mock control cells (23, 30, 31), authenticated and mycoplasma tested, were cultured in Dulbecco’s modified Eagle’s medium (D6429, Sigma Aldrich) supplemented with 10% FBS (F9665, Sigma Aldrich), 1% penicillin-streptomycin (P/S) and 1% blasticidin (D429, Sigma Aldrich). A day before ChIP, the media was changed to Dulbecco’s Modified Eagle Medium (DMEM)-phenol-red free with 1% charcoal-stripped fetal bovine serum (FBS, 12676011, ThermoFisher). Cell lines were not treated with E2 since previous studies has revealed that transduced ERβ functions ligand independent in CRC cell lines, possibly due to activation through growth factors and phosphorylations (23, 32, 33).

### p65 ChIP

For each ChIP experiment, 60x10^6^ cells were used. Cells were treated with TNFα (30 ng/ml, 11088939001, Roche, lot no: 25885600) Sigma-Aldrich) for 30 min and washed with PBS before cross-linking. Cells were first cross-linked with 2 mM disuccinimidyl glutarate (DSG) (20593, lot TF263080, Thermo Scientific) for 45 min during shaking. After washing (three times, with PBS) to remove DSG, they were cross-linked again with formaldehyde (1%) for 10 min during shaking. The double crosslinking was used to capture both short- and long-range p65 chromatin interactions. Glycine (final concentration 0.125M) quenched the cross-linking reaction. After washing (twice with PBS), cells were collected and pelleted by centrifugation. Cell pellets were further processed at 4°C using ice cold reagents. After lysis in LB1 (50Mm HEPES, 140mM NaCl, 1mM EDTA, 10% glycerol, 0.5% NP-40 and 0.25% triton-x) for 10 min, and centrifugation (4500 rpm, 5 min), pellets were suspended in LB2 (10 mM Tris-Hcl, 200 mM NaCl, 1 mM EDTA) for 5 min, centrifuged, and dissolved in LB3 buffer (10 mM Tris-Hcl, 100 mM NaCl, 1 mM EDTA, 0.5 mM EGTA, 0.1% Na-deoxycholate and 0.5% Na-lauroylsarcosine) to separate nuclear chromatin. Sonication generated 200-500 bp long fragments of chromatin. Following centrifugation (13000 rpm, 5 min), supernatants were collected in low-binding DNA tubes and incubated overnight with p65 antibody [Invitrogen, mouse monoclonal, cat no: 33-9900, lot no: QJ216251, RRID : AB_2533153, validated in (34)] or IgG (Santa Cruz, mouse polyclonal, cat no: sc-2025, lot no: J1514, RRID : AB_737182) as control. Next, samples were incubated with 30 µl protein G Dynabeads (cat no: 10004D, Invitrogen) for 3h. Beads were washed in sequential steps using TSE1 (20 mM Tris-HCl, 150 mM NaCl, 2 mM EDTA, 0.1% SDS and 0.1% Triton-X), TSE2 (20 mM Tris-HCl, 500 mM NaCl, 2 mM EDTA, 0.1% SDS and 1% Triton-X), LiCl buffer (20 mM Tris-HCl, 1 mM EDTA, 250mM LiCl, 1% NP-40 and 1% Na-deoxycholate) and TE buffer (10 mM Tris-HCl and 1 mM EDTA), and eluted (NaHCo3 (0.75%) SDS (1%), proteinase K (200 ng/µl)) overnight at 65°C, and finally treated with RNase A (1h at 37°C). QIAquick PCR purification columns (Qiagen, cat no: 28104) were used to purify DNA.

### ChIP-Sequencing

Libraries of the ChIP DNA were prepared and sequenced by the National Genomic Infrastructure (NGI) for Bioinformatics and Expression Analysis (BEA). DNA libraries were prepared using the NEB Next Ultra II DNA Library Prep Kit for Illumina (p/n NEB #E7645) and quality confirmed using TapeStation (DNA D1000 ScreenTape, Agilent). Libraries were loaded (1.8 pM end concentration of 1%) and sequenced (75 cycles, single read) using NextSeq 550 (Illumina).

### ChIP-Seq Data Analysis

Spliced Transcripts Alignment to a Reference (STAR) was used to map unique ChIP-Seq reads to the human reference genome assembly hg38 (GRCh38) with the alignIntronMax flag set to 1. Peak calling was performed using Hypergeometric Optimization of Motif Enrichment (HOMER) over input with a four-fold enrichment as cutoff and applying a false discovery rate (FDR) less than 0.001. Peaks which overlapped within 200 bp and were present in at least two out of three biological replicates were used for downstream analysis. Raw tag counts were normalized using R and binding pattern differences were identified with edgeR package. To cluster and visualize the different peaks, Complex heatmap from R was used. Promoter regions were defined as -1kb to +100bp from TSS and genomic distribution of binding sites were identified by HOMER. Gene functional annotation was performed using Database for Annotation Visualization and Integrated Discovery (DAVID), with P <0.05 considered as significant.

### Data Availability

The p65 ChIP-Seq data is deposited in the Gene Expression Omnibus (GEO) repository (GSE160856), TNFα bead array gene expression data was published previously (available at GSE65979), and SW480ERβ input and HT29ERβ input controls (GSE149979).

## Results

First, to understand the role of p65 transcriptional activity and oncogenic functions in CRC, we aimed to characterize its genome-wide binding in human CRC cells and correlate this to the TNFα-mediated transcriptional impact in the same cells. We used two well-characterized human colorectal adenocarcinoma cell lines: HT29 from a female primary tumor and SW480 from a male Dukes’ type B primary tumor.

### The p65 Cistrome of Colon Cancer Cells

**Figure 1:**
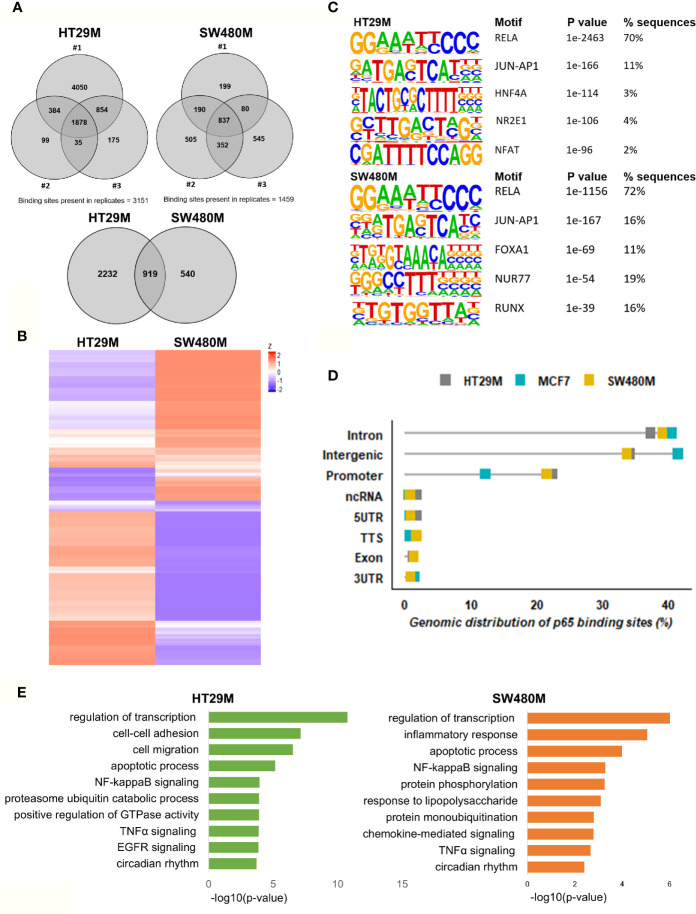
Genomic distribution of p65 binding sites in colon cell lines. **(A)** Identified p65 binding sites in three replicates of colon cancer cell lines HT29 and SW480 with those detected in at least two replicates used for further analysis and highlighted (top), and their overlap between cell lines (bottom), represented using Venn diagram. **(B)** Heatmap representing p65 binding sites in the two cell lines. **(C)** Motifs highly enriched in p65 binding sites identified by HOMER using *de novo* motif analysis and sorted by p-value. **(D)** Genomic distribution of p65 binding sites in relation to gene locations. **(E)** Biological functions enriched in genes nearest p65 binding sites (-50kb +2kb).

After optimizing the protocol for antibody specificity and including a double cross-linking (DSG-formaldehyde) procedure to capture long-range interactions of p65, we analyzed the chromatin bound by p65 in triplicate experiments of each cell line, HT29 and SW480. The sequencing produced between 24M and 65M (80%) of high-quality mapped reads per sample (**Table S1**). We identified a total of 12,504 (HT29) and 5004 (SW480) significantly enriched p65 peaks compared to input (**Table S1**). Out of these, 3151 and 1459 binding sites were found in HT29 and SW480 cells, respectively (**Figure 1A**). Whereas more p65 chromatin-binding sites were detected in HT29 cells overall, comparison between the two cell lines revealed that 63% (919) of sites found in SW480 were also found in HT29 (**Figures 1A, B** **)**. We next used HOMER to determine DNA motifs of the identified peaks. As expected, we identified RELA as the top motif, followed by JUN-AP1, in both cell lines (**Figure 1C**). This corroborates the specificity of the antibody and the protocol. Further, the transcription factors HNF4A and NFAT motifs were present in 3-5% of the HT29 binding sites, and FOXA1 and RUNX1 were relatively abundant (12% and 17%) in the SW480 p65 cistrome. Thus, we present the p65 cistrome of two different CRC cell lines and identify a shared common core, as well as cell-line specific differences.

### p65-Bound Genes Are Involved in Migration and Circadian Clock

**Figure 2:**
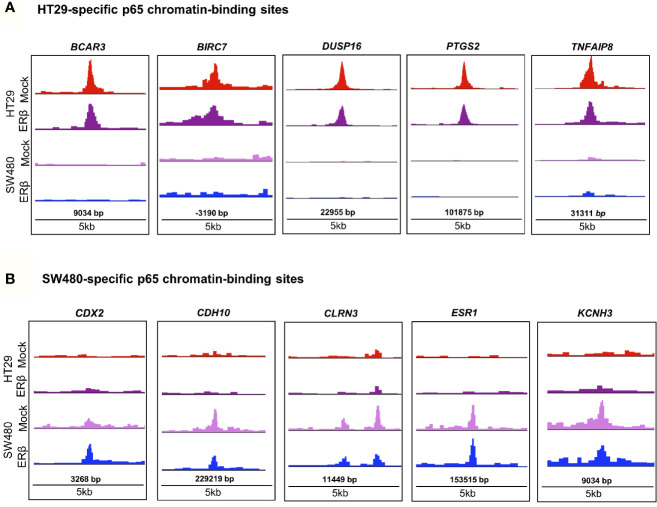
p65 chromatin binding sites. Peaks of p65 chromatin binding mapped to gene sequences in HT29 **(A)** and SW480 **(B)** cells using UCSC genome browser. Bp indicates distance from corresponding gene’s TSS.

In order to decipher how the above identified p65 binding may impact gene expression, we analyzed where the binding sites were located in relation to known genes. We found a highly similar pattern in both cell lines, with about 38% of sites located within introns, 34% in intergenic chromatin regions, and 22% within the promoter area (-1kb to +100bp from the transcription start sites, TSS) of genes (**Figure 1D**). The top-20 most enriched promoter sites in both cell lines include well-known p65 targets such as NFκB regulators *NFKBIB*, *NFKBIZ*, *TNFAIP3* (35), *BCL3* (36), and *BIRC3* (37, 38), NFκB subunits *NFκB2* (p52) and *RELB*, tumor suppressor p53, *TNIP1*, and *CREB1* (**Table S2**). p65-binding sites unique to either cell line also included well-known p65 target genes (HT29: *BCAR3*, *BIRC7*, *DUSP16*, *PTGS2*, and *TNFAIP8*; SW480: *CDX2*, *CDH10*, *CLRN3*, *ESR1* (ERα), and *KCNH3*, **Figures 2A, B** **)**. Pathway analysis of genes bound by p65 (-50kb to +2kb of TSS) revealed that genes with transcriptional regulatory functions (e.g. *JUND*), cell adhesion and migration (e.g. *WNT5B*, *BCAR1*, *TGFB1*, *CXCL16*), NFκB signaling, TNFα signaling, and apoptosis were enriched. This is in accordance with general NFκB functions (39, 40). We also identified a novel pathway, not previously associated with p65, including circadian rhythm in both cell lines (**Figure 1E**). Circadian rhythm genes bound by p65 included the central circadian regulator *CLOCK*, *BMAL2*, *CREB1*, and *KLF10*. In conclusion, we note highly concordant binding to cis-regulatory chromatin in proximity of genes within expected functions and further identify potential mechanism for p65 regulation of the colon circadian rhythm.

### p65 Recruitment Correlates With TNFα-Regulated Gene Expression in CRC Cells

**Figure 3:**
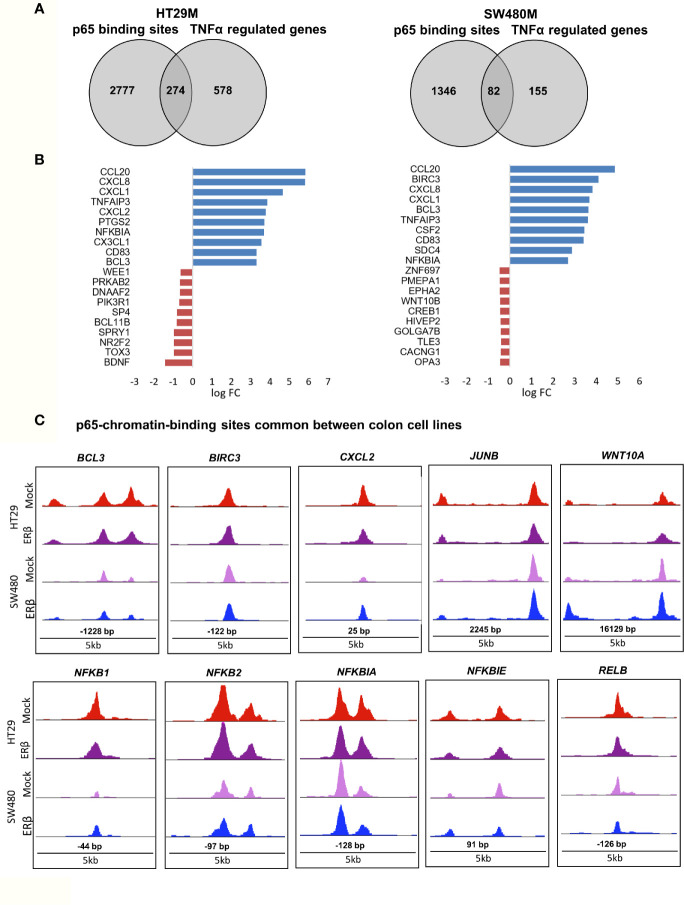
p65 transcriptional regulation in colon cell lines. **(A)** Number of genes with p65 chromatin binding sites and corresponding transcriptional regulation upon TNFα (10ng/ml, 2 h) treatment, per cell line. **(B)** The top-10 TNFα upregulated and downregulated genes with p65 binding sites in both cell lines (HT29, SW480). **(C)** Enrichment signal of p65 binding sites present in both cell lines, illustrated using UCSC genome browser.

While binding of a transcription factor indicates a potential gene regulation of nearby or distant genes, all such bindings do not translate into actual gene regulation. To determine the effect p65 binding has on transcriptional regulation of corresponding genes in CRC cells, we linked the p65 ChIP-Seq data to our previously generated TNFα (2-h treatment) transcriptome data set of the same cells (22). We found coordinated p65 binding and short-term TNFα regulation of 274 genes in HT29 and 82 genes in SW480 cells (**Figure 3A**, **Table S3**). Out of these, 59 genes (72% of those identified in SW480) were bound by p65 and regulated by TNFα in both cell lines (**Table S3**). All 59 core genes were upregulated by TNFα in both cell lines. Motif enrichment analysis of the p65-bound and TNFα-regulated core genes demonstrated significant enrichment for the p65 motif. Overall, the TNFα-regulated genes associated with p65-binding sites were mainly involved in the gene ontology functions of NFκB signaling, TNFα signaling, and inflammatory pathways. These included regulations and binding of known NFκB target genes (*BCL3*, *CCL20*, *CXCL1*, *CXCL8*, and *NFKBIA*, Fig 3B-C). Other known targets were differently bound and regulated in HT29 (*BCL11B*, *DUSP16*, *KLF6*, *RELA*) and SW480 (*IL23A*, *PRRG1*) cells (**Table S3**). In conclusion, this data clearly shows a strong transcriptional activity by p65 in both CRC cell lines, with p65-bound and regulated genes involved in critical CRC pathways, including apoptosis and cell migration.

### p65 Cistrome Differs Between Colon and Breast Cancer Cell Lines

**Figure 4:**
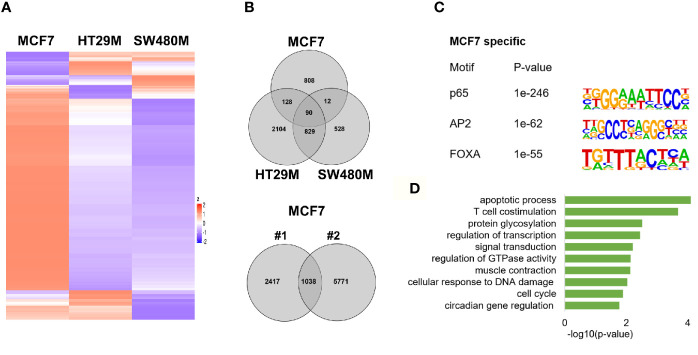
p65 cistrome in colon vs breast. **(A)** Heatmap illustrating p65 binding sites in breast (MCF7) and colon cancer cell lines (HT29, SW480). **(B)** Venn diagram comparing the p65 binding sites in MCF7, HT29, and SW480 cells. **(C)** DNA motifs located in MCF7-specific p65 binding sequences **(D)**. Pathways enriched among the gene ontology functions assigned to genes located nearest to MCF7-specific p65 binding sites.

To explore the extent that p65 binding is conserved between colon and breast cancer, we compared our generated p65 cistrome (colon) with previously published p65 ChIP-Seq data of the ER-positive breast cancer cell line MCF7. We selected a data set that also used double crosslinking (41). A heatmap illustrating p65 chromatin-binding sites in MCF7, HT29 and SW480 cells are shown in Fig 4A. Only 22% (230 sites) of MCF7 p65 binding sites were present in either CRC cell line (**Figure 4B**). Also, a markedly lower fraction of p65 sites were located by promoters in MCF7 cells (12% versus 21-22%, **Figure 1D**). The predominant motifs in MCF7 were NFκB (p65) itself, supporting accuracy of this data set, but were otherwise different (FOXA and AP2, **Figure 4C**) from colon (AP1, **Figure 1C**). The enriched biological functions for p65 sites specific for MCF7 also included apoptosis, transcription regulation, cell cycle, and circadian clock (**Figure 4D**). Thus, our study shows a cell specificity of p65 binding, where it binds different motifs and regulates different genes in different tissues or cell lines, but the biological functions of the regulated genes appear to have similar roles in cancer cell lines.

### ERβ Diminishes p65 Chromatin Binding in HT29 Cells

**Figure 5:**
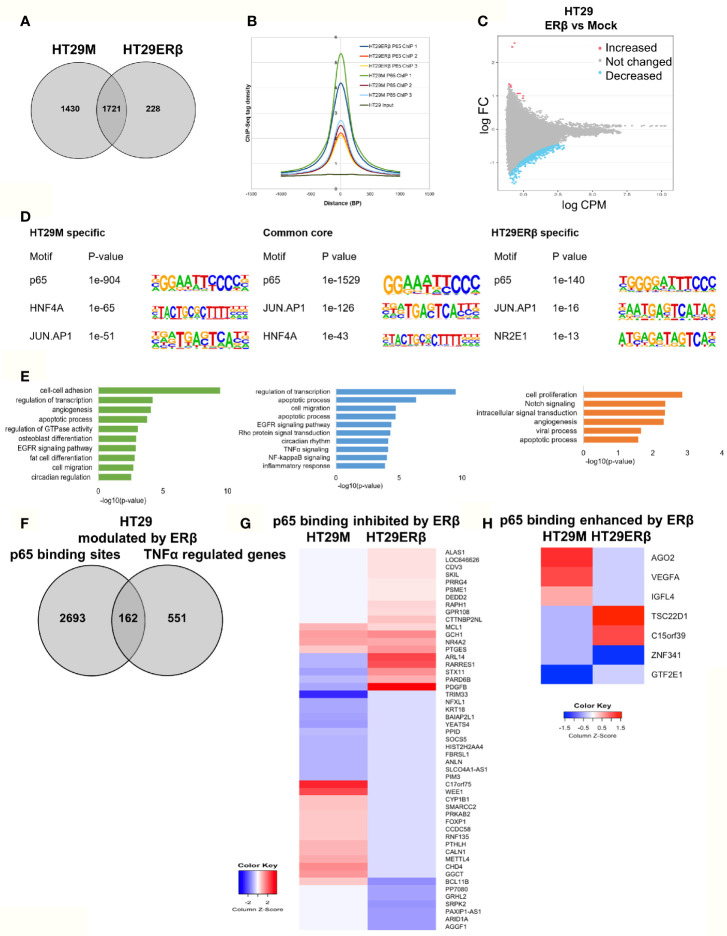
ERβ diminishes p65 chromatin binding in HT29 cells. **(A)** Venn diagram comparing p65 binding sites in HT29 cells with and without expression of ERβ. **(B)** Density plot representing the distribution of p65 tag densities in three replicates each of HT29 cells with and without ERβ. **(C)** Volcano plot highlighting statistically significant differences of p65 binding in HT29 cells in presence and absence of ERβ, using sliding window approach. **(D)** Motifs highly enriched in p65-bound sequencing in HT29 cells only in absence of ERβ, regardless of ERβ expression (core genes), and only in presence of ERβ, respectively. HOMER was used to identify genomic distribution and motifs of p65 binding sites across the genome. **(E)** Biological functions enriched among genes located nearest to p65 binding sites in HT29 cells depending on ERβ expression. **(F)** Overlap of genes located nearest to the p65 binding sites and those genes where ERβ expression impacted TNFα gene response in HT29 cells. **(G, H)** Heatmap representing ERβ modulation of the TNFα-regulated genes, of genes located nearest to p65 binding that was **(G)** inhibited and **(H)** enhanced by ERβ in HT29 cells. Z score values were calculated from the logarithmic fold changes, which represent a value’s relationship to the mean of a group of values. A positive Z score indicates the values above the mean and negative if it is below the mean.

Next, we aimed to study whether the mechanism whereby estrogen impacts inflammatory signaling in colon involves p65 chromatin binding. As we have previously found that ERβ can attenuate pro-inflammatory cytokine IL6 signaling in CRC cell lines (23) and regulate several important NFκB target genes and TNFα signaling *in vivo* (22), we explored whether ERβ impacts the p65 cistrome. We performed p65 ChIP-Seq in the same CRC cells, with and without (mock) expression of ERβ. In HT29, we found that whereas 1721 sites remained bound by p65 in both conditions, 1430 p65 binding sites were no longer detected in presence of ERβ. Further, a smaller fraction of 228 new binding sites were identified, only in presence of ERβ (**Figure 5A**). Using density plot, we noted that ERβ reduced the overall p65 binding in all three replicates (**Figure 5B**). We also analyzed this using a sliding window approach, with a window of 200 bp and calling for enriched regions between mock and ERβ, and identified the same trend (**Figure 5C**). Next, to investigate whether specific p65-binding motifs were affected by ERβ, we performed *de novo* motif analysis. The predominant p65 motifs in HT29 remained both in the absence and presence of ERβ (p65, AP-1, HNF4A, **Figure 5D**). Thus, the presence of ERβ reduced p65 chromatin binding and affected its distribution (numerous sites disappeared) but did not affect the type of motifs bound. Corresponding pathway analysis indicated that ERβ hindered p65 chromatin binding to genes with activities in cell adhesion, migration and circadian clock, while enabling binding by genes related to cell proliferation and Notch signaling (**Figure 5E**).

We also compared the p65 ChIP-Seq data with corresponding TNFα gene expression data, with and without ERβ (**Figure 5F**). We identified that 162 of p65-bound genes were regulated by TNFα differently in presence of ERβ. Among those, ERβ also inhibited p65 binding by 51 genes and enhanced binding of 7 genes. A heatmap illustrates how ERβ and resulting lack of p65 binding, affects TNFα-mediated regulation of these 51 genes (**Figure 5G**). Notably, presence of ERβ either inhibited TNFα-mediated response (50%), or enabled TNFα induction (31%) for the majority of these genes. These genes were mainly involved in functions such as negative regulation of transcription, negative regulation of cell proliferation, and chromatin remodeling. The seven genes where ERβ enhanced p65 binding were also impacted in terms of TNFα-mediated gene expression (**Figure 5H**). For example, ERβ enabled p65 binding and TNFα upregulation of *TSC22D1* and binding followed by downregulation of *ZNF341*, but blocked (presumably through p65 recruitment) TNFα-mediated upregulation of *VEGFA*, *AGO2* and *IGFL4*. We thus identified that ERβ reduces a sizeable fraction of p65 binding, modifying TNFα regulation in HT29 cells, especially for genes involved in e.g. cell proliferation and cell-cell adhesion.

### ERβ Enhances p65 Chromatin Binding in SW480 Cells

**Figure 6:**
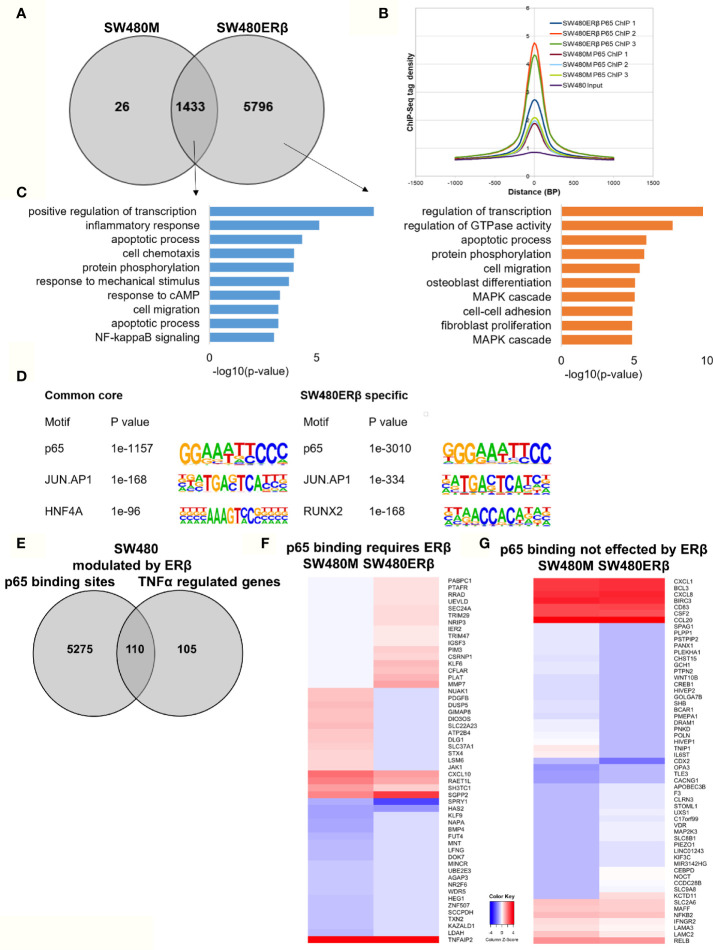
ERβ enhances P65 chromatin binding in SW480 cells. **(A)** Venn diagram of p65 binding sites in SW480 cells with and without expression of ERβ. **(B)** Density plot representing the distribution of p65 tag densities in three replicates each of SW480cells with and without ERβ. **(C)** Biological functions enriched among genes located nearest to p65 binding sites in SW480 cells depending on ERβ expression. **(D)** Motifs highly enriched in p65-bound sequencing in SW480 cells only in absence of ERβ, regardless of ERβ expression (core genes), and only in presence of ERβ, respectively. HOMER was used to identify genomic distribution and motifs of p65 binding sites across the genome. **(E)** Overlap of genes located nearest to the p65 binding sites and those genes where ERβ expression impacted TNFα gene response in HT29 cells. **(F, G)** Heatmap representing ERβ modulation of the TNFα-regulated genes, of genes located nearest to p65 binding that **(F)** required ERβ or **(G)** was not affected by ERβ. Z score values were calculated from the logarithmic fold changes, which represent a value’s relationship to the mean of a group of values. A positive Z score indicates the values above the mean and negative if it is below the mean.

Similarly, we explored the impact by ERβ on p65 chromatin binding in SW480 cells. Contrary to HT29, few binding sites were decreased upon introduction of ERβ and nearly all (1433 sites) remained. However, a high number (5796) of new p65 binding sites appeared in presence of ERβ (**Figure 6A**). This enhanced p65 binding was also evident for all replicates in the density plot (**Figure 6B**). Pathway analysis showed that p65 binding sites dependent on ERβ were located by genes involved primarily in transcription regulation, GTPase activity, apoptotic process, protein phosphorylation, cell migration, and MAPK cascade (**Figure 6C**, right panel). The p65 and AP-1 motifs remained highly enriched in SW480 ERβ, but we note that RUNX2 motifs were more common when ERβ was present (**Figure 6D**). RUNX2 is also upregulated by ERβ (23).

In terms of gene expression, ERβ modulated transcription of 110 TNFα-regulated genes that also had p65 binding sites in SW480ERβ cells (**Figure 6E**). About half of these (53 genes) had p65 bound by regulatory chromatin only in presence of ERβ. The corresponding TNFα-mediated gene regulation is illustrated in a heatmap (**Figure 6F**). Interestingly, ERβ (presumably by recruiting p65 to the chromatin) inhibited the TNFα activation of the majority of these genes (56%, or 30 genes). Another 16 TNFα-response genes required ERβ and resulting p65 binding for their induction (**Figure 6F**), including *DUSP5* [which regulates inflammatory gene expression of TNFα (42)], nuclear receptor *NR2F6*, and *KLF9*. Gene ontology enrichment reveals that genes within cell proliferation and cell migration were enriched among the p65 regulations modified by ERβ in SW480 cells. Despite the finding that ERβ enhanced p65 binding in SW480 cells, the resulting transcription of these genes was mostly inhibited. ERβ also modulated expression of p65-bound TNFα-regulated genes, without impacting p65 chromatin binding (57 genes). Among these, expression was attenuated in most (24), and enhanced in some (14). Genes suppressed by ERβ included *BCL3*, *BIRC3*, *CCL20*, *NFκB2* and *RELB* (**Figure 6G**), all of which are associated with poor prognosis in CRC (43–47).

Thus, ERβ clearly modulates p65 binding and TNFα response also in SW480 cells. ERβ appeared to enhance p65 binding, but still repress TNFα transcriptional activity.

### ERβ Modulates p65 Signaling in Colon Cells

**Figure 7:**
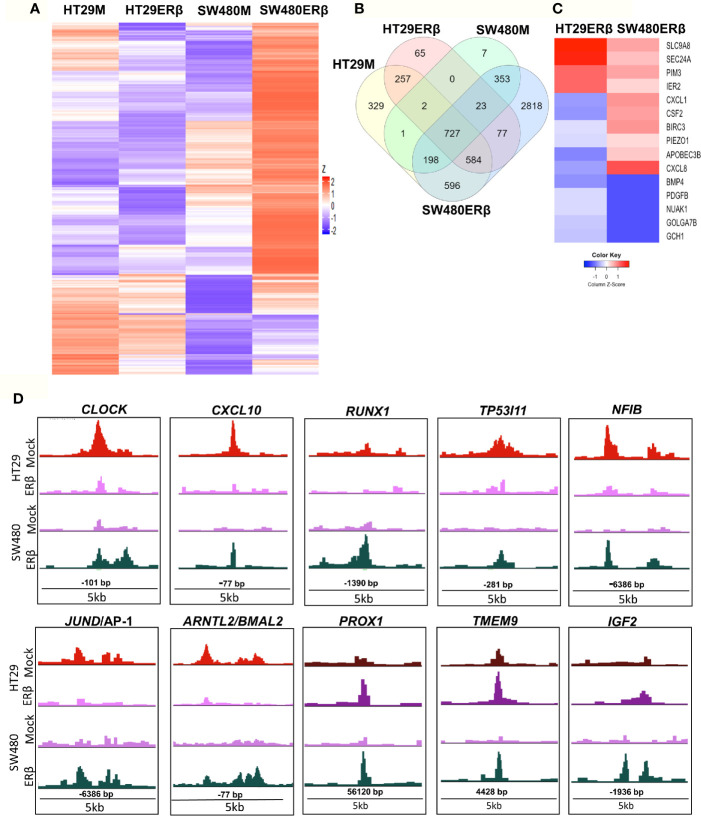
ERβ influences P65 chromatin binding in colon cancer cells. **(A)** Heatmap representing p65 chromatin binding over the genome in absence or presence of ERβ, in HT29 and SW480 cells. **(B)** Venn diagram comparing p65 chromatin binding sites in HT29, SW480 cell lines with and without ERβ. **(C)** Heatmap illustrating the impact of ERβ on TNFα modulated genes with identified p65 chromatin binding sites in HT29 and SW480 cells, respectively. Z score values were calculated from the logarithmic fold changes, which represent a value’s relationship to the mean of a group of values. A positive Z score indicates the values above the mean and negative if it is below the mean. **(D)** Examples of p65 chromatin binding sites that was identified only in presence of ERβ in SW480 cell lines, but bound the same sites in HT29 in absence of ERβ and p65 chromatin binding sites enhanced by ERβ in both HT29 and SW480 cells illustrated using the UCSC genome browser.

From the above findings, we conclude that ERβ impacted p65 binding in both CRC cell lines, partly in different ways but with similar outcome in terms of TNFα-mediated gene regulation. Here, we compare p65 chromatin binding (ChIP-Seq data) between the two cell lines, with and without ERβ (**Figure 7**). A heatmap illustrates the reduced p65 binding upon ERβ expression in HT29, and the enhancement noted in SW480 (**Figure 7A**). A Venn diagram comparing p65 binding in the four conditions (HT29 and SW480 with and without ERβ, **Figure 7B**) shows that a large fraction of p65 sites (727 sites) are bound in all conditions, and 77 sites are enhanced by ERβ in both HT29 and SW480. ERβ appears to enable p65 binding 56 kb downstream of TSS of *PROX1* in both cell lines (**Figure 7D**). PROX1 is a transcription factor highly upregulated in colon cancers and previously shown to be regulated by ERβ post-transcriptionally through miR-205 (21). Overall, 15 genes were bound by p65 and regulated by TNFα, both in absence and presence of ERβ, in both cell lines (**Table S4**). Out of these, the TNFα response of 12 genes was modulated in opposite direction by ERβ in HT29 and SW480 cells, which include important target genes such as *BIRC3, CXCL1, CXCL8* and *PDGFB* (**Figure 7C**). We further identified 596 p65 binding sites opposed by presence of ERβ in HT29 but, in contrast, bound only in presence of ERβ in SW480 cell line (**Figure 7B**). This core set of genes includes the well-known p65 interacting protein AP-1, NFIB, and circadian clock genes (*CLOCK, CXCL10, RUNX1, TP53I11, NFIB, BMAL2/ARNTL2*, **Figure 7D**). Altogether, these results indicate that in addition to conserved patterns there are also considerable cell specific differences in p65 binding between HT29 and SW480 cells, and that ERβ impacts the p65 cistrome and TNFα response in both cell lines.

## Discussion

Activation of NFκB in the intestinal epithelia can lead to colitis-induced CRC (48). Our study attempts to understand the molecular mechanisms behind the CRC promoting role of inflammation, by studying the cistrome of p65 and investigating how this is impacted by ERβ. We characterize the p65 genome-wide chromatin binding in two different CRC cell lines, and specify similarities and differences. We find that presence of ERβ impacts p65 binding and corresponding TNFα-mediated transcription.

Our work emphasizes that NFκB binds primarily through RELA and JUN-AP1 motifs in cells with a colon origin. Genes nearest to p65-bound chromatin were involved in inflammatory response, cell proliferation, cell migration, and, interestingly, circadian clock (*BMAL2*, *CLOCK*). A previous study has shown that dysregulation of circadian rhythm increases the risk for colorectal cancer (49). Indeed, *CLOCK* gene mutations have been identified in 53% of CRC with microsatellite instability (MSI) (50). Moreover, another study identified that mutations in the CLOCK1 gene increased the risk of developing CRC (51). In breast cancer cells, several studies identified a link between circadian genes and NFκB signaling pathway (52, 53). In colon cancer, two studies have shown that REV-ERB-α through NFκB modulates circadian clock and reduced DSS-induced colitis (54, 55). In our recent studies, we have showed that ERβ can modulate the impact of TNFα-NFκB activity in CRC cell lines and *in vivo* using the AOM-DSS mouse model (22). We have also demonstrated that intestinal ERβ regulates the expression of the circadian clock gene *Bmal1* (*Arntl1*) in colon of HFD-fed mice (56). Here, in addition to identifying p65-binding sites, we demonstrate that the activation of the TNFα-NFκB axis impacts the expression of circadian genes. Moreover, we show that ERβ interferes with the general p65 chromatin binding, including the circadian genes *CLOCK* and *BMAL2* (**Figure 7C**). Taken all this into account, our interpretation is that p65 modulates circadian genes in the colon in the pro-inflammatory pro-tumorigenic condition, and that ERβ can change this and thereby oppose the inflammatory condition that drives development of colon cancer.

Interestingly, p65 chromatin binding appears relatively distinct between the two CRC cell lines, in support with the fine-tuned cell-specific manner whereby NFκB controls transcriptional regulation. The cell lines are indeed different in several respects. While both are derived from primary colon adenocarcinomas, the HT29 cell line is derived from a likely pre-menopausal (44-year-old) woman, whereas SW480 originates from a 50-year-old man (57, 58). We have also reported sex differences in the non-tumor and tumor transcriptome of CRC patients, which impacted biomarker discovery (59). The different female-male origin of the cell lines used here, may indeed impact the different regulation of p65 cistrome or its modulation by ERβ. However, further studies are needed to clarify this. Moreover, HT29 cells are CIMP (CpG island methylator phenotype) positive, and SW480 cells are CIMP negative. Aberrant methylation of the CpG islands has been shown to impact chromatin binding and accessibility to transcription factors (60, 61). Their mutational profile also differs, with HT29 having mutations in BRAF (V600E), PIK3CA (P449T), and p53 (R273H), and SW480 in KRAS (G12V) and p53 (double mutant alleles R273H and P309S, however still retaining functionality of many p53-associated pathways) (62, 63). These proteins are important transcriptional regulators that can also influence the binding of transcription factors (64, 65). In parental CRC cell lines HT29 and SW480, neither ERα nor ERβ is expressed, while MCF7 cell line expresses ERα, which has been shown to interact with p65 (24). These factors may all modulate the p65 cistrome.

While the p65 binding pattern was similar between the two CRC cell lines, the p65 cistrome of breast cancer cell line MCF7 was more distinctly different. One of the well-known interaction-partner of p65 is the p53 protein (65). Recently, it was shown that mutant p53 enhances NFκB activity in mice, leading to chronic inflammation and associated CRC (65). Another study demonstrated that p53 mutants directly interact with NFκB in SW480 cells (15). MCF7 cell line has wild type p53, whereas both HT29 and SW480 cells express the R273H p53 mutant protein, which inhibits DNA binding (66, 67). Hence it is possible that the p53 status impacts p65 cistrome in these cell lines, and further studies are needed to explore this hypothesis.

We have previously shown that TNFα triggers a transcriptional response in both CRC cell lines, and that ERβ modulates this (22). Here, we correlate the transcriptional response with p65 chromatin binding sites, and how ERβ modifies the p65 cistrome. To be noted, in order to optimize experiments, different treatments times and concentrations of TNFα were used in ChIP-Seq (30 ng/ml, 30 min) and for transcriptional analysis (10 ng/ml, 2h). Further, the transduced ERβ is expressed at higher levels in SW480 compared to HT29 (1.8 times more), and previous data suggests that TNFα may increase transactivation of ERβ (22). These factors may all influence the kinetics of the mechanisms described here, but are not expected to have a major influence on the mechanism per se. Our findings offer mechanistic underpinnings of how inflammation modulates specific signaling pathways and how ERβ can attenuate cytokine-induced carcinogenic response in CRC cells.

The strength of this study includes the genome-wide approach to decrypt these interactions, which together with the validated high-quality ChIP-Seq data generates unbiased and reliable data. The significance of these findings is reinforced by the use of two different CRC cell lines and the comparison with transcriptional impact, as well as comparisons between our results and published data generated from cells of other origin. A shortcoming includes the use of exogenous expression of ERβ in the CRC cell lines. However, cell lines mostly lack endogenous expression of ERβ (21, 22). Further, it may be preferable to use non-tumor colonic cell lines, as one aim of the study was to investigate how p53 can prevent CRC through its impact on NFκB signaling. However, a key interest was also to decipher the oncogenic NFκB signaling in CRC, and along with the lack of suitable non-tumor cell lines at hand, and our access to highly characterized cell lines with exogenous expression of ERβ, this is the balance we chose. Further, the difference of antibodies used between our study and the breast cancer tissue study (41), may contribute to the differences found.

In conclusion, we provide a mechanistic foundation for a better understanding of how estrogen influences inflammatory signaling through NFκB in CRC cells.

## Data Availability Statement

The p65 ChIP-Seq data is deposited in the Gene Expression Omnibus (GEO) repository (GSE160856), TNFα bead array gene expression data was published previously (available at GSE65979), and SW480ERβ input and HT29ERβ input controls (GSE149979).

## Author Contributions

CW contributed to conceptualization. Methodology performed by RI, LH, and AA. Validation done by RI and LH. Formal analysis was done by RI and LH. Curation, RI. RI and CW wrote the first original draft and all authors commented or edited. Visualization, RI. Supervision, CW and AA. Project administration, CW. All authors contributed to the article and approved the submitted version.

## Funding

National Cancer Institute at the National Institutes of Health (R01CA172437), the Swedish Cancer Society (CAN2015/591, CAN2018/596), Swedish Research Council (2017–01658), and Region Stockholm (HMT grant 20170804). RI was supported by a PhD student grant (KID) from the Karolinska Institutet.

## Conflict of Interest

The authors declare that the research was conducted in the absence of any commercial or financial relationships that could be construed as a potential conflict of interest.

## Supplementary Material

Supplementary Table 1 ChIP-Seq statistics.

Supplementary Table 2 p65 promoter binding sites in colon cancer cells.

Supplementary Table 3 p65-bound and TNFα-regulated genes in colon cancer cells.

Supplementary Table 4 Differently modulated TNFα-regulated genes by ERβ in colon cancer cells.
